# Control of Pyrethroid-Resistant Chagas Disease Vectors with Entomopathogenic Fungi

**DOI:** 10.1371/journal.pntd.0000434

**Published:** 2009-05-12

**Authors:** Nicolás Pedrini, Sergio J. Mijailovsky, Juan R. Girotti, Raúl Stariolo, Rubén M. Cardozo, Alberto Gentile, M. Patricia Juárez

**Affiliations:** 1 Instituto de Investigaciones Bioquímicas de La Plata (CCT La Plata CONICET-UNLP), Facultad de Ciencias Médicas, Universidad Nacional de La Plata, La Plata, Argentina; 2 Coordinación Nacional de Control de Vectores, Córdoba, Argentina; 3 Instituto de Patología Experimental, Facultad de Ciencias de la Salud, Universidad Nacional de Salta, Salta, Argentina; 4 Coordinación de Gestión Epidemiológica, Ministerio de Salud Pública, Salta, Argentina; Liverpool School of Tropical Medicine, United Kingdom

## Abstract

**Background:**

*Triatoma infestans*-mediated transmission of *Tripanosoma cruzi*, the causative agent of Chagas disease, remains as a major health issue in southern South America. Key factors of *T. infestans* prevalence in specific areas of the geographic Gran Chaco region—which extends through northern Argentina, Bolivia, and Paraguay—are both recurrent reinfestations after insecticide spraying and emerging pyrethroid-resistance over the past ten years. Among alternative control tools, the pathogenicity of entomopathogenic fungi against triatomines is already known; furthermore, these fungi have the ability to fully degrade hydrocarbons from *T. infestans* cuticle and to utilize them as fuel and for incorporation into cellular components.

**Methodology and Findings:**

Here we provide evidence of resistance-related cuticle differences; capillary gas chromatography coupled to mass spectrometry analyses revealed that pyrethroid-resistant bugs have significantly larger amounts of surface hydrocarbons, peaking 56.2±6.4% higher than susceptible specimens. Also, a thicker cuticle was detected by scanning electron microscopy (32.1±5.9 µm and 17.8±5.4 µm for pyrethroid-resistant and pyrethroid-susceptible, respectively). In laboratory bioassays, we showed that the virulence of the entomopathogenic fungi *Beauveria bassiana* against *T. infestans* was significantly enhanced after fungal adaptation to grow on a medium containing insect-like hydrocarbons as the carbon source, regardless of bug susceptibility to pyrethroids. We designed an attraction-infection trap based on manipulating *T. infestans* behavior in order to facilitate close contact with *B. bassiana.* Field assays performed in rural village houses infested with pyrethroid-resistant insects showed 52.4% bug mortality. Using available mathematical models, we predicted that further fungal applications could eventually halt infection transmission.

**Conclusions:**

This low cost, low tech, ecologically friendly methodology could help in controlling the spread of pyrethroid-resistant bugs.

## Author Summary

Chagas disease, also known as American Trypanosomiasis, is the most relevant parasitic disease in Latin America, being a major burden that affects mostly poor human populations living in rural areas. The kissing-bugs of the Triatominae family transmit the parasite *Trypanosoma cruzi* by infectious blood-sucking; *Triatoma infestans* is the vector of major relevance in the southern Cone of South America. Current control strategies, heavily based on residual insecticide spraying, are threatened by the emergence of pyrethroid-resistant bug populations. Furthermore, ensuring the long-term and sustainable control of this overwhelming disease remains a major challenge. Here we show the utility of a simple, low-cost, biological control methodology against *T. infestans* bugs, regardless of their susceptibility to pyrethroid insecticides. It is based on the understanding of the initial contact interactions between a mycoinsecticide agent—the fungus *Beauveria bassiana*—and the host defense barrier, the bug cuticle. The proposed methodology is also supported by present data showing a relationship between the triatomine cuticle width and its hydrocarbon surface components, with insecticide resistance. These results will help to provide a safe and efficient alternative to overcome pyrethroid-resilience of these noxious bugs. A high transfer potential to immediate application in rural communities located in remote areas inaccessible to sanitary control teams, and to the control of other Chagas disease vectors as well, is also envisaged.

## Introduction

Chagas disease is currently the most important parasitic disease of the Americas representing a serious health and social burden for most Latin-American countries, with current estimates of about 10 million people infected and 40 million at risk [1]. The causative agent of this disease, *Trypanosoma cruzi*, is mostly acquired through contact with infected blood-feeding triatomine bugs that usually infest poorly constructed dwellings, hiding in thatched roofs and cracks in the walls. Control of *Triatoma infestans*, the major disease vector in the Southern Cone area of South America, is mostly based on the use of chemical insecticides, particularly pyrethroids [2]. In the last decade however, different levels of pyrethroid resistance have been detected in Bolivia, the site of *T. infestans* origin and dispersal [3], and in some neighboring areas of Argentina [4]. In other disease vectors, reduced penetration, increased sequestration, metabolic resistance, and alterations in the target site and behavior modification, are amongst the most widely recognized mechanisms of resistance [5].

The insect cuticle is the first barrier against the penetration of contact insecticides; it is also the entry portal of entomopathogenic fungi. The epicuticle is the outermost surface layer; in triatomines it is covered by very long chain hydrocarbons (HC) and a variety of lipids [6]; the surface HC fraction accounts for the major protective barrier role [7]. Entomopathogenic fungi have the ability to fully degrade insect cuticular hydrocarbons and to utilize them for energy production and incorporation into cellular components [8]. Fungal adaptation to grow on an insect-like HC medium can effectively improve virulence by an enhanced HC catabolism [9]. In other alkane-degrading microorganisms, the first reaction of the catabolic pathway is catalyzed by a cytochrome P450 monooxygenase (P450alk) [10]. Several P450alk genes revealed overlapping but differential expression after fungal growth on insect-like HC (Pedrini, unpublished data). Enzymes involved in the last steps of insect HC degradation were also induced in HC-grown fungi [11],[12].

The efficacy of entomopathogenic fungi to control malaria mosquito had focused interest on similar alternatives for other vector-borne diseases, with the additional benefit of low risk to the development of fungal resistance [13],[14]. Several fungal strains of *Beauveria bassiana* and *Metarhizium anisopliae* virulent to Chagas disease vectors are already known [15]–[17].

The aim of this research was to characterize the relationship between *T. infestans* cuticular layer and pyrethroid resistance, and to investigate the potential enhancement of *B. bassiana* virulence by adaptation to grow on insect-like hydrocarbons. We also explored a practical vector control approach based on manipulating bug behavior in order to facilitate contact with *B. bassiana*. This methodology would be particularly useful to control pyrethroid-resistant Chagas disease vectors.

## Materials and Methods

### Study design

- First generation insects from different geographic locations were used for pyrethroid resistance evaluation, together with cuticular hydrocarbon content and cuticle thickness analyses.
- *B. bassiana* virulence against *T. infestans* was evaluated in laboratory bioassays.
- An attraction trap based on CO_2_ release was assayed in experimental houses.
- An attraction-infection device, combining the CO_2_-based trap and the fungus, was assayed both in experimental houses and rural human dwellings.
- We also estimated the potential *T. cruzi* transmission risk index after intervention with *B. bassiana*-based traps.

### Insects

Collection sites for *T. infestans* are detailed in Table 1. First laboratory generation insects from pyrethroid-susceptible (Py-S) populations collected in rural villages of the Chacabuco department, Chaco province, Argentina (27° 14′ S, 61° 12′ W; 85 masl), and pyrethroid-resistant (Py-R) domestic populations from Salvador Mazza department, Salta province, Argentina (22° 03′ S, 63° 41′ W; 804 masl) were reared at the Universidad Nacional de Salta for pyrethroid resistance measurement; lots of the same colony were also submitted to the Instituto de Investigaciones Bioquímicas de La Plata (INIBIOLP) for hydrocarbon analyses and bioassays. To calculate the hydrocarbon amount per insect weight and surface, bugs were weighed using a micro analytic Mettler balance, and their surfaces were estimated as the sum of a semi ellipsoid and an ellipse surface; the larger and minor radium were estimated as half of the bug length and width, respectively. First generation insects from Campo Largo, Salta province, Argentina (22° 05′ S, 63° 56′ W; 929 masl), and from Tierras Nuevas, Tarija department, Bolivia (21° 44′ S, 63° 56′ W; 645 masl) were also used for pyrethroid resistance evaluation. First laboratory generation of Py-S *T. infestans* specimens also came from bugs collected in the San Martin department, San Luis province, Argentina (32° 23′ S, 65° 40′ W; 921 masl). Insects were reared both at the Coordinación Nacional de Control de Vectores (CNCV) for the assays in experimental houses, and at the INIBIOLP for laboratory bioassays. Insects were reared at 28°C, 50–60% relative humidity, and fed weekly on chickens.

**Table 1:** Insect origin and assays performed.

Country	Province/State	County	Assay
Argentina	San Luis	San Martín	Laboratory bioassays Field assays (experimental houses)
	Chaco	Chacabuco	Pyrethroid resistance measurement Cuticular hydrocarbon analyses Laboratory bioassays
	Salta	Salvador Mazza	Pyrethroid resistance measurement Cuticular hydrocarbon analyses Laboratory bioassays
	Salta	Campo Largo	Pyrethroid resistance measurement Field assays (rural village houses)
Bolivia	Tarija	Tierras Nuevas	Pyrethroid resistance measurement Field assays (rural village houses)

### Measurement of resistance to pyrethroid insecticides

Deltamethrin resistance was tested in first-instar nymphs [18]. Five deltamethrin doses were assayed, within an appropriate mortality (M) range (0%<M<100%) 24 hr after topical application of 0.2 µl of each solution on the dorsal abdomen. Each dilution was tested by triplicate (10 insects/each). Doses giving 50% lethality (LD_50_ values) values were estimated by probit analysis [19], with a 95% confidence interval.

### Insect lipid extraction and fractionation

Epicuticular lipids were extracted by immersing recently freeze-killed fifth-instar insects (14-d old, one week after a blood meal; 8 replicates of 3 insects each), both from Py-S and Py-R colonies, in three successive portions of hexane (6 ml/g insect) for 3 min each, and analyzed by thin layer chromatography (TLC) [20]. Then, internal lipids were obtained by whole insect homogenization and extraction with chloroform: methanol (2∶1) after partitioning with water (4∶1). Exuviae lipids were obtained from fourth-instar molts after immersion in hexane for 24 hr. Hydrocarbons were separated from other lipid components by adsorption chromatography, and analyzed by capillary gas chromatography (CGC) using a Hewlett-Packard 6890 gas chromatograph (Hewlett Packard, Wilmington, USA) equipped with a split/splitless injection port, operated in the splitless mode and fitted with a non-polar fused silica (0.25 mm) DB-5 capillary column (30 m×0.32 mm I.D.). Oven temperature was programmed from 50°C (hold time 2 min) to 180°C at 20°C/min, then 180°C to 310°C at 3°C/min (hold time 10 min). The flame ionization detector (FID) was held at 320°C [21]. The HC amounts were estimated by co-injection with known amounts of *n*-tetracosane. Standards of 24 to 44 carbons (Sigma, St. Louis, USA) were run in similar conditions to estimate the response factor for each hydrocarbon peak. For CGC-mass spectrometry analyses, the CGC equipment was coupled to an Agilent mass selective detector MS 5975C VL (Agilent Technologies, Wilmington, USA) operated at 70 eV, chromatographic conditions as above. Mass spectral data was analyzed as previously described [22].

### Scanning electron microscopy

Cuticles were excised from 9 Py-R and 9 Py-S fourth-instar bugs, and then frozen in liquid N_2_ in order to help obtaining undistorted transversal cuts by cryofracture. The cuticle fragments were then sputter-coated with gold, mounted directly without dehydration, and examined in a JEOL JSM 6360 VL equipment (JEOL Ltd., Tokio, Japan). The width of the cuticle layer was estimated in the second tergite, taking at least two measures per cuticle, using the ImageJ 1.4g software (Rasband WS, National Institute of Health, Bethesda, USA).

### Fungal strain and cultivation

The *Beauveria bassiana* strain GHA (Laverlam International, Butte, USA) used in this study was kindly provided by Dr Stefan Jaronsky (USDA ARS NPARL, Sidney, USA). Fungi were maintained on complete medium agar (CMA) plates containing 0.4 g KH_2_PO_4_, 1.4 g Na_2_HPO_4_, 0.6 g MgSO_4_.7H_2_O, 1.0 g KCl, 0.7 g NH_4_NO_3_.7H_2_O, 10 g glucose, 5 g yeast extract, and 15 g agar in 1000 ml of distilled water. Glucose-grown (GG) fungi were obtained by culturing on CMA at 26°C for 14 d. Assays were also performed on CMA lacking glucose and yeast extract (deficient medium agar, DMA), and supplemented with the synthetic hydrocarbon *n*-octacosane (*n*-C28) (Sigma, St. Louis, USA), as previously described [16]. Briefly, the hydrocarbon (2.5 ml of a 10% hexane solution, w/v) was layered onto the surface media and the solvent was evaporated. Fungi already grown on CMA were incubated at 26°C on hydrocarbon-enriched DMA for other 14-d period, to obtain the hydrocarbon-grown (HCG) cultures.

### Fungal dry formulation

Two formulations based on dry conidia and diatomaceous earth (DE) mix [23] (2∶1, w∶w) were used. DE came from Perma-Guard Inc. (Albuquerque, USA). Formulation 1: the conidia harvested after cultivation in agar (GG or HCG fungi) were dried at 25°C in a glass desiccator containing silica gel beads, and mixed up with DE. The resulting powder was grinded by passing through a coffee mill. Formulation 2: The conidia:DE mix was obtained employing the commercial powder formulation of conidia (1.27×10^11^ conidia/g, 98% viable). At the dose used, DE produced no mortality.

### Laboratory fungal bioassays

#### Insect mortality and median lethal time

Individual Py-S insects (14-d old fourth-instar nymphs, 1 week after a blood meal) were immersed for 6 s in fungal suspensions of each culture condition (GG and HCG). Different concentrations (3×10^7^, 2×10^8^, and 1×10^9^ conidia ml^−1^) in distilled water containing 0.01% Tween 80, were assayed. Then, insects were placed on separate 250-ml plastic containers covered with a muslin cloth, and maintained afterwards at 26°C, 50% relative humidity, and without feeding. Control insects were immersed in water containing Tween 80 (0.01%). A similar treatment (2×10^8^ conidia ml^−1^) was applied to Py-R. Five replicates of each treatment (GG, HCG, and controls) were performed in one experiment, with 10 insects per replicate. A second experiment was similarly performed five months later, using a different generation of insects. Mortality was checked daily during 3 weeks. Dead insects were placed in individual humid chambers to confirm fungal infection. Median lethal time (MLT) was estimated as Σ (days_n_×dead nymphs_n_)/total dead nymphs [24].

#### Horizontal transmission assay

Laboratory assays were performed to evaluate the potential horizontal transmission (autodissemination) of conidia from previously contaminated Py-S insects to healthy insects (third-, fourth-, and fifth-instar). Nine nymphs (3 insect/instar) were artificially contaminated by allowing in contact for 5 min in a 15-cm Petri dish; the dish surface was covered with the formulation 1. Then, the “contaminated” insects were released together with non-contaminated specimens (9 insect/instar) into a plastic container (50×30×20 cm), pieces of pleated corrugated cardboard (8×3 cm) were placed in each corner as shelter. Insects were maintained during 1 month at rearing conditions without feeding; then, dead insects were collected and fungal infection was evaluated as described above. Survivors were fed on chicken once and maintained in individual containers to further check for mortality and developmental cycle. Control groups were treated similarly except that the Petri dish contained no conidia. For each instar, mortality was calculated as N×100/Nt, where: N: number of dead nymphs by autodissemination, Nt: total nymphs exposed to “contaminated” insects. The initially “contaminated” bugs were not considered for mortality estimation.

### Field assays

#### Assays in experimental houses

Experimental houses (‘ranchos’) were used at the CNCV experimental field station located in Santa María de Punilla, Córdoba province, Argentina (31° 14′ S, 64° 28′ W; 952 masl). The experimental ranchos (1×1×1 m) were built with similar elements as those used in the lowest economic resource rural dwellings, the walls were made of sun-dried mud-bricks (‘adobe’), the floor was plain soil and the roofs were made of ‘jarilla’ shrub (Larrea sp.) and mud, covered with a piece of plastic (1.2×1.2 m) to protect from rain. To prevent insect escape, an aluminum structure with mosquito net sides completely surrounded each experimental house. Py-S first generation fifth-instar insects 2-weeks old and unfed after molt were used. Assays were performed in March (average monthly values of 20.4°C temperature and 77.0% r.h) and December 2006 (average monthly values of 22.9°C temperature and 68.0% r.h).

A volatile attraction trap consisted in a CO_2_ source set into a 500 mL container with a 3-cm diameter opening covered with a cloth net. The CO_2_ source was obtained by alcoholic fermentation of *Saccharomyces cereviciae* using dried commercial baker yeast (10 g in 1-L sucrose aqueous solution, ð = 1.050 g/cm^3^) providing at least 10 ml/min gas flow for 24 h. The container was completely buried on the floor, except for the opening inserted centrally in a piece of cardboard (15×12 cm) fully covered with a double side sticky band. Thirty bugs were released into each experimental house. Same number of control houses was similarly set, except that no baker yeast was added. A plastic cover fixed on the traps prevented insects to be eventually trapped during a 30-min initial period, time enough for insects to fully hide in the adobe bricks. After that period, the traps were quickly uncovered. Insects attracted and trapped with the sticky band were counted as positive 24 h after starting the assay.

An attraction/infection trap was also designed and tested; it consisted in a combination of the former trap contained within a cardboard box (17×14×5 cm) with the powdery formulation 1. Assays were performed similarly as described for the attraction trap. To allow insect entrance into the attractive box, 2 rectangular holes (2.5×0.6 cm) were made in two opposite sides of the box, at ground level. One gram of each fungal formulation (1×10^8^ con/mg, either of GG or HCG cultures) was set within the trap, 4cm-side square around the container. The experimental houses were totally dismantled 4 d after starting the assay, dead and alive insects were collected, and transported to the laboratory in individual vials. Cadavers were assessed in a humid chamber to confirm fungal infection.

#### Assays in rural village houses

The bioinsecticide performance against Py-R *T. infestans* populations was tested in two field sites next to the Argentina-Bolivia border, previously detected by the local sanitary officers as heavily infested and resistant to pyrethroid treatment; the last professional insecticide spray was performed 4 months before present experiments. The first experiment was performed in Tierras Nuevas, Yacuiba, Tarija department, Bolivia, in April 2008 (average monthly values are 20.6°C temperature and 78.0% r.h) (Fig. S1A). Nine houses were selected based on vector presence, construction materials, and distribution within a 2 km width area. The selected houses were made of adobe or mud-stick (‘palo a pique’), indoor walls were unplastered, and roofs were made of locally available shrubs, finished with a layer of soil and metal pieces. All dwellings had a floor made of flattened dirt. The number of inhabitants per house ranged from three to nine humans in 1–2 bedrooms; dogs (2–3/house) were reported to sleep outside. Peridomiciliary structures were chicken coops and corrals. Another field assay was carried out in the rural village of Campo Largo, Acambuco, Salta province, Argentina, 50 km southwest from the former (Fig. S1A). Experiments were performed in June 2008 (average monthly values are 14.6°C temperature and 67.4% r h). Five out of total 14 houses in the village, with 1 room/house (∼3m×3m×2.5m) were selected as described for the former site. The whole area belongs to the northwest ending of the subtropical rain forest (‘yunga’), the fauna and flora went through a recent (25 years) and dramatic ecological change as result of unrestrained deforestation.

In both field sites, attraction/infection trap devices similar to those described for the experimental station were assayed, using the formulation 2. The attraction CO_2_ source was replaced after 48 h thus providing at least 3–4 d of volatile attractant release. Prior to the assays, a two-person team searched by sight for triatomine presence in room areas, household goods, and beds (one man-hour/house); no flushing out treatment was applied in order to avoid physical damage or affecting the behavior of the bugs. At this stage, no bug collection was performed.

Either attraction-infection or attraction traps were set at “treated” or “control” houses, respectively. The traps were both set on the walls, close to the insect refuges detected (4 traps/room), and on the floor (2 traps/room). Two weeks after starting the assay, a 3-person team thoroughly searched for dead and alive insects inside the traps and in their surroundings. Dead and alive bugs collected were stored separately in labeled containers and submitted to the INIBIOLP to confirm fungal infection. Insects collected from “control” houses were used as controls; no *B. bassiana* infection was detected. Due to the sanitary emergence by the presence of Py-R bugs, and the restricted accessibility roads, only “treated” houses were allowed in Campo Largo.

### Ethics statement

This study was performed within the guidelines established by the Bioethic Regulations, and with the approval, of the Public Health Ministry of Salta province in Argentina. Given that there is no local institutional review board in the Bolivian region under study, the Bolivian authorities of the Health Department Service (SEDES) of Tarija department, and the Secretary of Human Development of Tarija prefecture, received both written and oral information fully detailing the research protocol, and provided oral consent for the study, according to usual procedures in the region. The SEDES chief-officer participated as observer, and one technical officer of the Vector Control Program at Yacuiba (SEDES, Tarija) participated in the whole field assay procedure. Before the study, the objectives and the study protocol were explained during meetings with the community leaders of Campo Largo and Tierras Nuevas, and health professional and technical staff. Each head of family of these illiterate populations provided oral consent to perform the assay, after receiving the same information in the presence of local Vector Control Program officers, who participated as mediator. Oral consent was documented by a spreadsheet.

### Data analysis

Statistical significance of mortality and MLT data obtained from laboratory and experimental house assays was determined by Student's *t*-test, the significance level was set at *P*<0.05. To predict the effect of the trap and kill method on making smaller the number of bugs, and hence on parasite infection transmission, we estimated the potential *T. cruzi* transmission risk index (TcTRIp), defined as the maximum number of risky bites a human can receive per night [25]. Although actual number of bites on humans depends on the presence of other domestic blood sources [26], they were not included in the estimation.
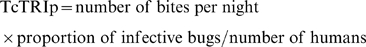



The number of bites per night was estimated according to [25] as:
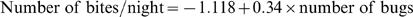



The relationship between total bug density and the number of infective bugs was estimated according to [27] as:
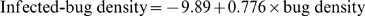



The average number of humans/house was 5. Both, the infected bug density and the number of bites per night were determined in about 40 houses of small rural infested villages (Santiago del Estero and Córdoba provinces, Argentina), of similar construction characteristics as those described in present study [25]. The three early nymph stages were excluded from the total bug density estimation because of their relatively low infection rates [28].

## Results/Discussion

Resistance ratio values [29] were quite close between Tierras Nuevas (79.7) in Bolivia, Campo Largo (99.3), and Salvador Mazza (106.1) in Argentina (Table 2), and with previous reports in the area [4].

**Table 2:** Toxicity of deltamethrin against first-instar *T. infestans* from Campo Largo and Salvador Mazza (Salta, Argentina) and Tierras Nuevas (Tarija, Bolivia).

Origin	n	Slope±SE	LD_50_ ng/insect (95% CL)	RR (95% CL)
Chacabuco*	180	1.47±0.22	0.24 (0.16–0.36)	–
Tierras Nuevas	150	1.38±0.25	19.5 (12.8–32.9)	79.7 (48.6–145.7)
Campo Largo	150	1.05±0.19	26.1 (15.4–57.5)	99.3 (45.7–216.1)
Salvador Mazza	150	1.43±0.25	30.2 (19.7–56.0)	106.1 (62.3–181.2)

RR: resistance ratio, calculated according to [29].

*: Reference population.

### Hydrocarbon content and cuticle thickness in pyrethroid-resistant and pyrethroid-susceptible *Triatoma infestans*

**Figure 1:**
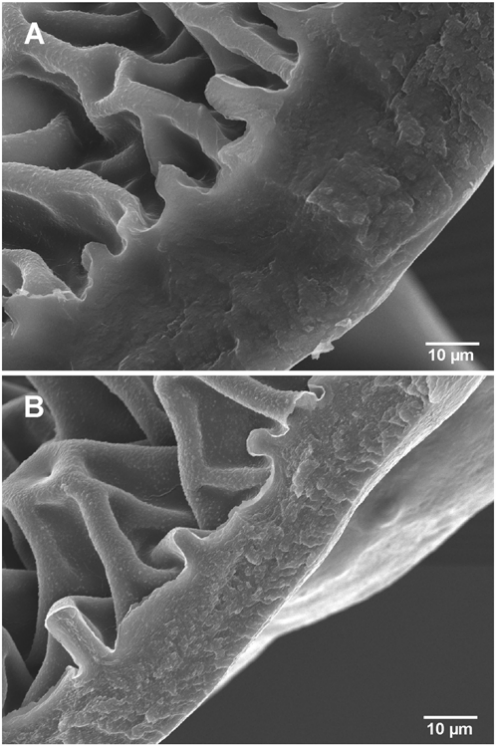
The cuticle of *T. infestans* fourth-instar nymphs. Scanning electron micrograph (SEM) of a transversal cut of the second tergite of pyrethroid-resistant (A) and pyrethroid-susceptible (B) bugs. The cuticle width was estimated in 32.1±5.9 µm and 17.8±5.4 µm respectively, *P*<0.0001. Magnification: 1,400×.

Mass spectral analyses revealed no qualitative difference between hydrocarbon components in Py-R and Py-S insects [30]. Because Py-R insects showed consistently lower weight and smaller size compared to Py-S insects (Table 3), we calculated HC amounts per unit weight or size (surface area). Both the epicuticular HC amount per insect weight (w/w) and the total HC amount per insect weight (w/w) were significantly higher (56.2±6.4% and 41.1±9.5%, respectively; *P*<0.03) for Py-R compared to Py-S insects (Table 3). Also, the epicuticle/total HC ratio was significantly higher (33.9±0.3%; *P*<0.002) for Py-R compared to Py-S insects, suggesting a selective HC transport to the surface. In a similar fashion, significantly higher (*P*<0.0002) HC amounts of Py-R compared to Py-S insects (43.5±2.2%) were extracted from the fourth-instar exuviae shed after molt to fifth-instar. Even at similar epicuticular HC amounts per bug, Py-R insects benefited with a smaller surface area to distribute them on, showing significantly higher (*P*<0.03) HC amount (29.2±9.6%) per unit surface than Py-S insects. Insect cuticle hydrocarbons participate in the regulation of the interaction with contact chemical and biological agents [31]. HC-free cuticles of *T. infestans* were more permeable to chemicals penetration than intact cuticles [7]; the enriched-HC surface layer very probably participates by restricting deltamethrin penetration in Py-R insects. Furthermore, scanning electron microscopy revealed that the cuticle of Py-R insects was significantly thicker than that of Py-S insects (32.1±5.9 µm and 17.8±5.4 µm respectively, *P*<0.0001) (Fig. 1). Recently, in a large scale microarray analysis to monitor gene expression in resistant and susceptible houseflies, genes possibly involved in thickening of the cuticle showed the most striking up-regulation [32]. A penetration factor (pen) was shown to contribute to low order resistance, although it can add significantly to the overall resistance mechanism when combined multiplicatively to increased detoxification and decreased target site sensitivity [33].

**Table 3:** Epicuticular and total hydrocarbon amounts in pyrethroid-resistant and pyrethroid-susceptible *T. infestans.*

Pyrethroid susceptibility	Fifth-instar nymph				Fourth-instar exuviae
	EHC/weight (ng/mg)	THC/weight (ng/mg)	EHC/THC	EHC/surface (ng/mm^2^)	THC (µg)/exuviae
Py-R	50.7±16.8	394.2±93.5	0.12±0.02	6.1±1.1	4.3±0.5
Py-S	32.5±11.9	279.4±83.4	0.09±0.01	4.7±1.2	3.0±0.3

Py-R: pyrethroid-resistant.

Py-S: pyrethroid-susceptible.

EHC: epicuticular hydrocarbons.

THC: total hydrocarbons. Bulk of THC is represented by the large internal hemolymph reservoir and the integument tissue; their amount is positively correlated to body weight [20].

Values are means of 8 replicates±SD of 3 insects each, and 7 replicates±SD of four exuviae each. In all columns, the difference between Py-R and Py-S values is significant (*P*<0.05).

The mean total weight of Py-S insects (170.8±65 mg) was significantly higher than that of Py-R nymphs (97.4±41 mg). Mean total surface estimated for Py-S insects (837±160 mm^2^), was significantly different (*P*<0.05) than that of Py-R insects (683±98 mm^2^).

### Fungal virulence evaluation in laboratory assays

#### Effect of the carbon source

We first compared the effect of the carbon source used for fungal growth on the virulence against Py-S bugs. Infection was achieved by immersion in conidia suspensions, at different doses. At relatively low dose producing well below 100% mortality, the killing power of hydrocarbon-grown (HCG) fungi was significantly higher than that of glucose-grown (GG) fungi (71.1±16.0% and 46.7±16.3%, respectively; *P*<0.03). At higher doses, attaining ∼100% mortality for both culture conditions, the speed to kill was significantly higher for HCG fungi (*P*<0.05), as shown by the MLT values of 6.4 – 5.3 d compared to 8.0 – 6.3 d for GG fungi, at increasing doses, respectively (Table 4).

**Table 4:** Virulence of *B. bassiana* grown in different carbon sources against fifth-instar *T. infestans* nymphs under laboratory conditions.

Dose (con/ml)	Growth condition	Mortality (%)	Median lethal time (days)
3×10^7^	GG	46.7±16.3	8.9±1.2
	HCG	71.1±16.0*	8.9±2.1
2×10^8^	GG	90.0±17.0	8.0±0.3
	HCG	90.0±7.1	6.4±0.5*
1×10^9^	GG	100	6.3±0.3
	HCG	100	5.3±0.3*

GG: glucose-grown fungi.

HCG: hydrocarbon-grown fungi.

Values are means±SD. Two experiments of 5 replicates each (10 bugs/replicate) were performed.

*: The difference between HCG and GG values is significant (*P*<0.05).

#### Susceptibility of pyrethroid-resistant insects

**Figure 2:**
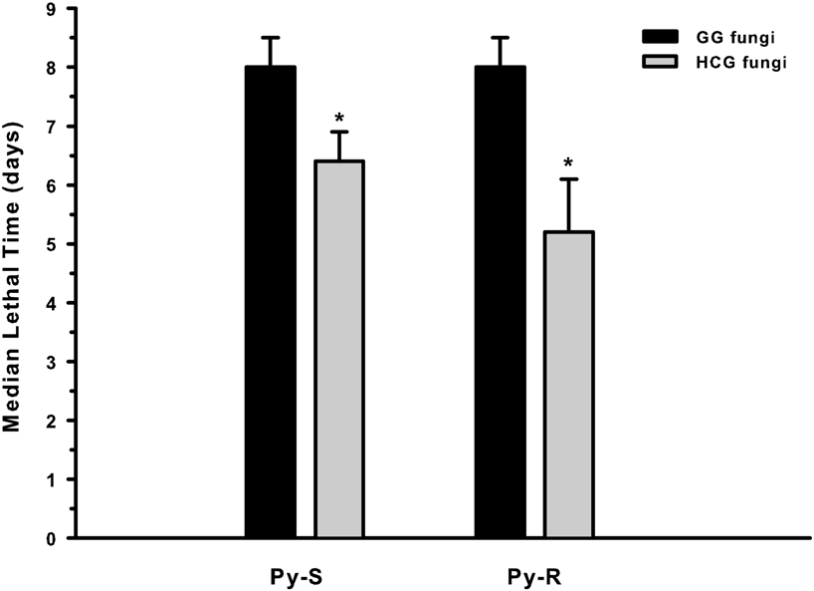
Median lethal time of both pyrethroid-susceptible (Py-S) and pyrethroid-resistant (Py-R) *T. infestans* treated with *B. bassiana* grown on two different carbon sources, under laboratory conditions. Fifth-instar nymphs were immersed for 6 seconds in a fungal suspension (2×10^8^ con/ml). Values are means±SD. Significant differences (*P*<0.05) between glucose-grown (GG) and hydrocarbon-grown (HCG) fungi are shown with an asterisk.

The susceptibility to fungi was compared between Py-R and Py-S insects. An early juvenile stage (3^rd^-instar) showed to be significantly (P<0.04) less vulnerable to fungal infection than the final (fifth) nymphal stage. Comparing the performance of GG fungi at a fixed dose (2×10^8^ con/ml), mortality values achieved were 79±15% (Py-S) and 72±15% (Py-R) for 3^rd^-instar nymphs, reaching to >90% for fifth-instar nymphs, both for Py-R and Py-S bugs. Using HCG fungi (same dose), mortality values of Py-R 3^rd^-instar bugs were significantly increased (P<0.03) to 90±9%; although not significant, a similar trend was observed for Py-S bugs (93±10%). Coincidently, the alkane-growth adaptation helped to reduce the MLT values of Py-R, from 8.0±1.7 d to 6.6±1.2 d, close to those of Py-S bugs. At higher mortality rates for the last instar bugs, a significant reduction in the MLT values was achieved with HCG fungi, both for Py-R (P<0.01) and Py-S (P<0.0004) last instar bugs (Fig. 2). Cuticle thickening and the higher surface HC content of Py-R bugs might be related to a reduced penetration of the pyrethroid insecticide, and thus contribute to decrease the effective dose of insecticide; on the contrary, these differences do not seem to affect the fungal contact and penetration through the cuticle.

#### Fungal horizontal transmission

**Figure 3:**
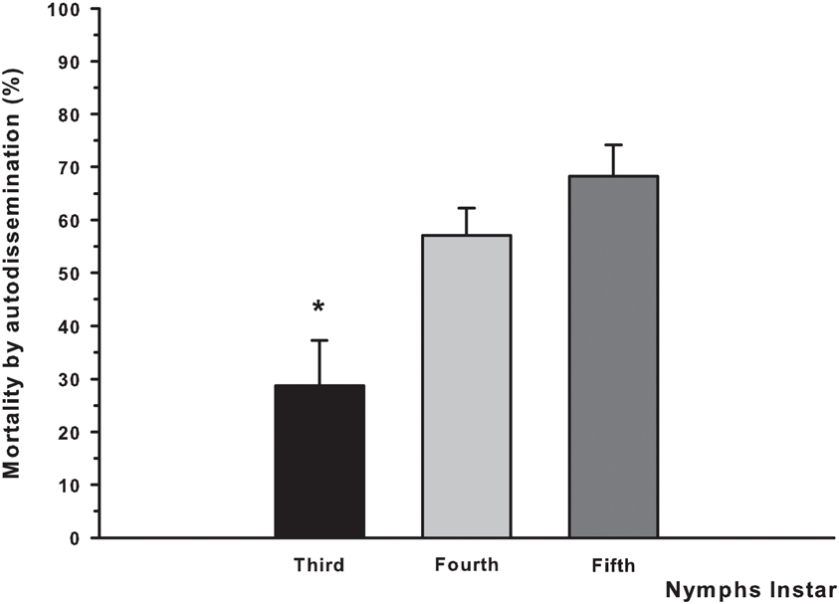
Horizontal transmission (autodissemination) of conidia from previously contaminated nymphs to initially non-infected nymphs. Three replicates were performed per experiment, and the experiment was repeated three times at bimonthly periods. The asterisk show significant differences (*P*<0.05). At the end of the experiment, only 3.7% of the fungus-treated survivors were able to molt, whereas 28.2% of the control bugs did molt.

We evaluated whether horizontal transmission (autodissemination) of the pathogen adds substantially to overall infection. We found that 50.9% of the originally non-infected bug population died after 30 d (Fig. 3). No mortality was detected for the control insects during the period tested. The characteristic bug aggregation behavior at resting sites [34] probably contributed to the autodissemination process. The significantly (*P*<0.05) lower success of autodissemination in third-instar bugs compared to late stages might be explained by a combination of stage selective susceptibility and a noticeable lower exposed surface per insect. Acceptable virulence parameters do not assure sustainable success of biopesticides in the field; viability loss and manipulation costs are among major concerns for the most usual mode of application by spraying. Some of these restrains can be overcome by using dry conidia formulations, shown to be reliable for the control of other disease vectors in field trials [35]. To take full advantage of the potential transmission achievable by conidia autodissemination, and hence reducing largely the amount of bioinsecticide required and manipulation complexity, it is feasible to manipulate the target insect behavior by means of attractant cues leading them toward specific inoculation sites (attract-infect devices) [36].

### Experimental field assays

Triatomine bugs exhibit characteristic daily locomotive and resting periods, during which they either actively search for a blood meal, or aggregate and rest in secluded refuges [34],[37]. Furthermore, blood-feeders are known to perceive the volatile secretions of their target hosts; among them exhaled CO_2_ is the main and widespread long range attractant [38],[39]. In *T. infestans,* the response level is ∼300–400 ppm above the threshold level in the laboratory [40].

#### Attraction trap

Pyrethroid-susceptible fifth-instar nymphs were released into experimental adobe houses (1×1×1 m) containing a CO_2_ source centrally located and surrounded by a double-side sticky band (attraction traps). Figure S2A shows the trap location. The proportion of bugs trapped with the sticky band was 58±11% after 24 h (Table 5); most of the attracted bugs (70%) were trapped in the first 6 hours (Fig. S2B). No bugs were trapped in the control houses; on the contrary, they remained hidden between the brick layers.

**Table 5:** *T. infestan*s response to either attraction or attraction-infection traps in experimental houses.

Trap	Fungi	CO_2_	Attraction (%±SD)	Mortality (%±SD)
Attraction	-	yes	58.3±11.1	0
	-	no	0	0
Attraction-infection	GG	yes	-	27.0±8.9
	HCG	yes	-	46.0±8.0

GG: glucose-grown *B. bassiana*.

HCG: hydrocarbon-grown *B. bassiana*.

Fifth-instar nymphs (Py-S) were released into experimental houses (30 insects/house) either for attraction or attraction-infection assays. The number of replicates varied from 3 to 9.

Attraction traps: a yeast solution (CO_2_ source) was poured within a plastic container centrally located and surrounded by a double-side sticky band (Fig. S2A and S2B). Control houses contained no CO_2_ source. Attraction was measured after 1 d.

Attraction-infection traps: Attraction traps (without the sticky band) including a dry fungal formulation placed on paper strips (Fig. S2C). After 4 d, houses were dismantled and dead and alive insects were collected. Mortality was evaluated after 14 d. Attraction traps (no fungi added) were used as controls.

#### Attraction-infection trap

A dry fungal formulation was incorporated in the trap (attraction-infection trap). Either GG or HCG conidia were deposited inside the trap, all around the CO_2_ source. Four days later, the experimental houses were fully dismantled; all the bugs were collected and kept separately to evaluate mortality and infection. Ten days later, the number of insects dead by fungal infection was significantly different (P<0.05), accounting for 27.0±8.9% (GG fungi) and 46.0±8.0% (HCG fungi) of the bugs attracted to the trap (Table 5 and Fig. S2C). No mortality was detected in the control traps.

### Field assays in rural villages

**Figure 4:**
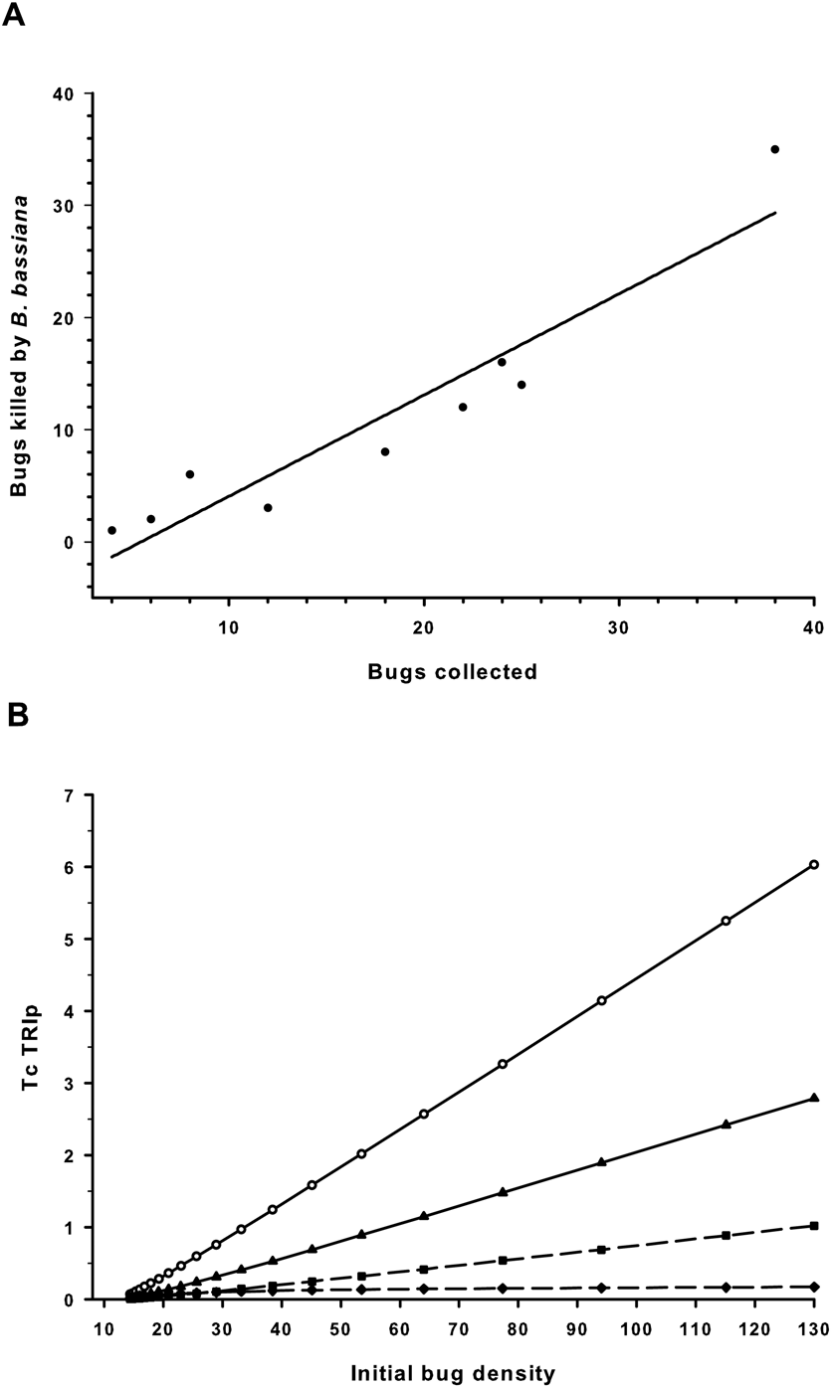
Efficacy of fungal intervention in rural villages, and estimation of bug population reduction on potential transmission risk. A) Relationship between the number of insects collected and the number of insects killed by *B. bassiana* after the intervention. The field experiment was performed in 9 houses from two rural villages in the Argentina/Bolivia border (Fig. S1A). Insects were collected manually, no chemical-trapping methodologies were used in order to avoid potential alterations in the process of fungal infection, and therefore they account for a small number of the actual catchable bug population. Adults were 40.3±7.0% of the total insects collected; 57.1±10.4% of the adults, and 55.1±10.4% of the nymphs were killed by fungi. Most of the nymphs collected (67.6%) belonged to fourth- and fifth-instar. These stages, along with adults, account for almost all bugs that are infected [28]. Linear regression: y = −4.98+0.90 x; r = 0.95. B) Estimation of potential *T. cruzi* transmission risk index (TcTRIp) after intervention with *B. bassiana*-based traps at village level. Solid lines show the predicted TcTRIp [25] without intervention (circles), and after bug population reduction due to the experimental fungal infection shown in A) (triangles). Dotted lines indicate the predicted values of TcTRIp after 2 (squares) or 3 (diamonds) hypothetical successive interventions. Initial bug density corresponds to an arbitrary number of insects prior intervention.

A field study was carried out in 2 neighbouring rural villages in an area infested with Py-R *T. infestans*, along the Argentina-Bolivia border (Fig. S1A). We evaluated the performance of the “attraction-infection” traps set on the walls and ground in selected houses (Fig. S1B). Dead and alive insects were collected 20 d after installing the traps, ranging from 4 to 38 bugs/house (Table 6); 52.4±7.7% of the bugs collected died by fungal infection (Fig. 4A). No *B. bassiana* infection was detected in the bugs from control houses. The number of insects collected does not allow discriminating mortality due to direct contact or horizontal transmission. However, at increasing number of bugs per house (during the warm season), it would be expected a higher contribution of fungal autodissemination to mortality. Dissemination of insect pathogens by attracting the target insect pest population into a “pathogen concentration site” has been successfully applied in the control of the African trypanosomiasis vector *Glossina sp.* in the field [41]. Even though a significant loss of viability under the sunlight and/or high humidity was expected, a good number of conidia were reported to be viable for a period of 20–30 d [42]. In our assays, viability of the conidia dry formulation was 88±3% after 3 months under field conditions.

**Table 6:** Field assays in rural villages.

		Treatment			Insects dead by fungal infection^c^	
Field site	House	Fungi	Attractant	Number of insects collected^b^	Number	Percentage
Tierras Nuevas, Bolivia	1	+	+	24	16	66.7
	2	+	+	4	1	25.0
	3	+	+	38	35	92.1
	4	+	+	12	3	25.0
	5	- a	+	6	0	-
	6	-	+	10	0	-
	7	-	+	4	0	-
	8	-	+	8	0	-
	9	-	+	6	0	-
Campo Largo, Argentina	10	+	+	8	6	75.0
	11	+	+	18	8	44.4
	12	+	+	25	14	56.0
	13	+	+	22	12	54.5
	14	+	+	6	2	33.3

a Attractant traps were placed in control houses (no fungi added).

b Dead and alive insects were collected in control and treated houses, 2 weeks after starting the assay.

c Dead insects were set in humid chambers to confirm fungal infection.

### Modeling the effect of bug population reduction on potential transmission risk

Considering bug population as the sole entomological factor, we simulated the eventual impact of bug population reduction on the risk of acquiring the parasite infection by estimating the parasite transmission potential risk index [25] using available estimations on the relationship between density of domiciliated bugs and the number of *T. cruzi* infected bugs [27]. Actual domestic bug densities are expected to be well above the number of insects detected in this study. Given an unlimited blood supply, the number of bugs per household has been estimated >300 bugs [26]. For an intermediate number (i.e., 100 bugs), the maximum number of risky bites per human per night (TcTRIp) could be reduced from 5.2 to 2.4 after one bioinsecticide application (Fig. 4B). A second bioinsecticide application could drop infection risk down to 0.88 bites per human per night, and further decline (0.16) after a third intervention. Comprehensive mathematical models to assess population dynamics of triatomine insects as well as *T. cruzi* infection transmission models are available, based on environmental, demographic and other factors estimation [26],[43]. However, no specific attempts were addressed on estimating the effect of bug number reduction on the risk of acquiring Chagas disease. The reduction in bug density can be increased and extended in time by simple procedures, either employing higher conidia doses, improved formulations, new attractant components, optimizing trap number, design, and location, in addition to regularly replacing attractants and conidia formulation.

### Final remarks

The results of this study may help to develop a new strategy to control Chagas disease vectors, particularly addressed to two scenarios: to control Py-R domiciliated bug populations, as here shown; it is also envisaged the potential application at the peridomiciliary level, where current tactics and procedures are reported to fail [44]. This simple and inexpensive methodology might be easily implemented at the local level; both the low-tech fungal mass production as well as the trap installation, including attractant re-filling and monitoring, in collaboration with sanitary agents from the community.

## Supporting Information

Figure S1 A) Map of the study area showing the location of *B. bassiana*-treated houses in the villages of Tierras Nuevas (Bolivia) and Campo Largo (Argentina). Both sites were infested with deltamethrin-resistant *T. infestans* populations (Table 2). B) Field assay settings at a rural village in the Chaco region. A typical rural dwelling selected for fungal application.
(2.39 MB TIF)

Figure S2 Experimental field assay. A) Experimental house. The arrow shows the attraction trap location. B) Bugs catched on a sticky surface during a 6-h period exposure to the CO_2_ source. The bottle containing the yeast suspension is buried on the floor; the central opening is covered with muslin and located at the floor level. C) Bugs killed in the “attraction-infection” device; here the CO_2_-releasing bottle is covered with a perforated lid. Only the bottom side of the trap is shown in the picture.
(2.12 MB TIF)
