# Supplementary figures and images for: Control of Pyrethroid-Resistant Chagas Disease Vectors with Entomopathogenic Fungi
Source: PLoS Negl Trop Dis. 2009 May 12;3(5):e434. doi: 10.1371/journal.pntd.0000434 (PMC2674565; doi:10.1371/journal.pntd.0000434)

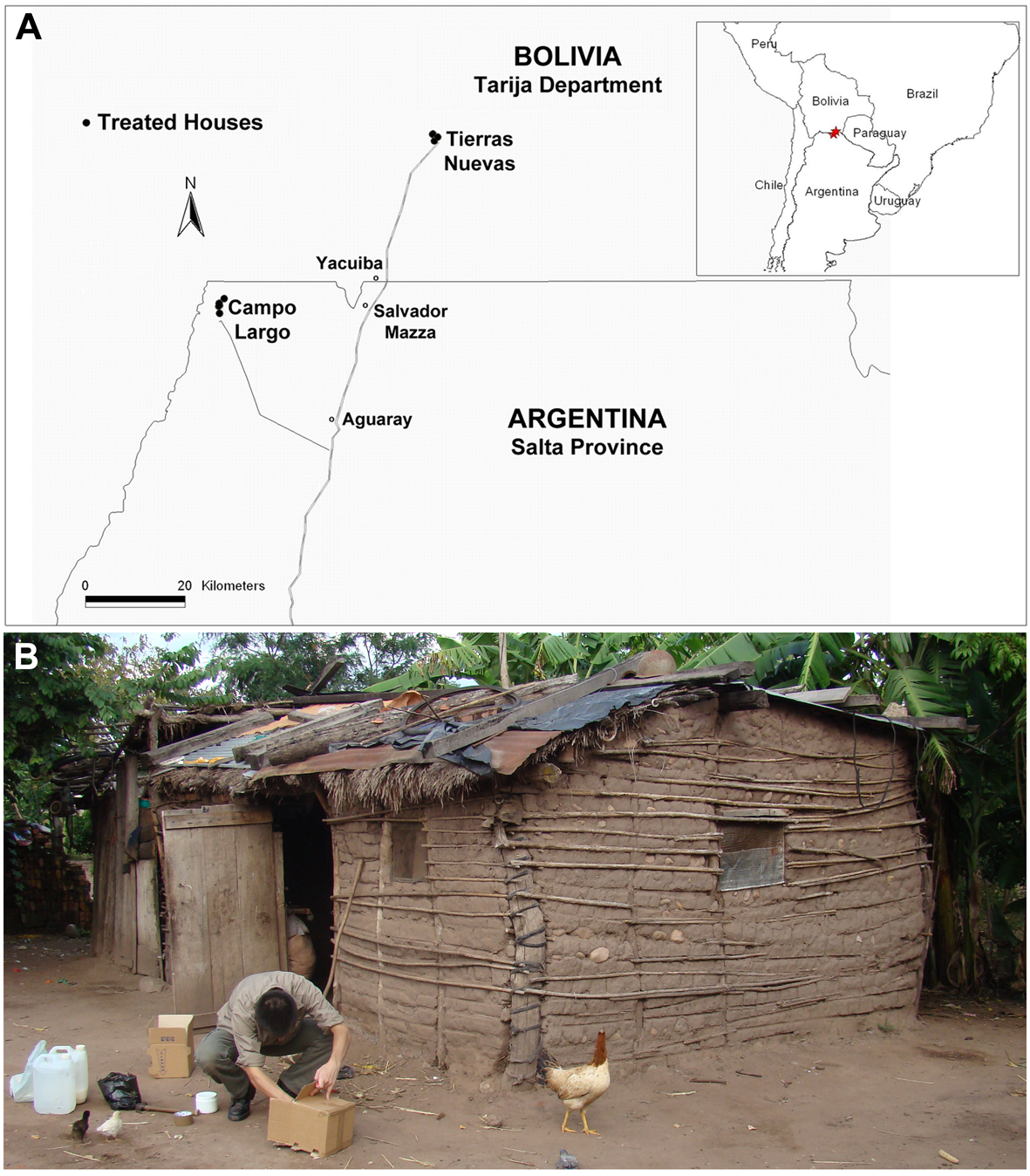

Supplement: Figure S1 — A) Map of the study area showing the location of B. bassiana-treated houses in the villages of Tierras Nuevas (Bolivia) and Campo Largo (Argentina). Both sites were infested with deltamethrin-resistant T. infestans populations (Table 2). B) Field assay settings at a rural village in the Chaco region. A typical rural dwelling selected for fungal application. (2.39 MB TIF) [file pntd.0000434.s001.tif]

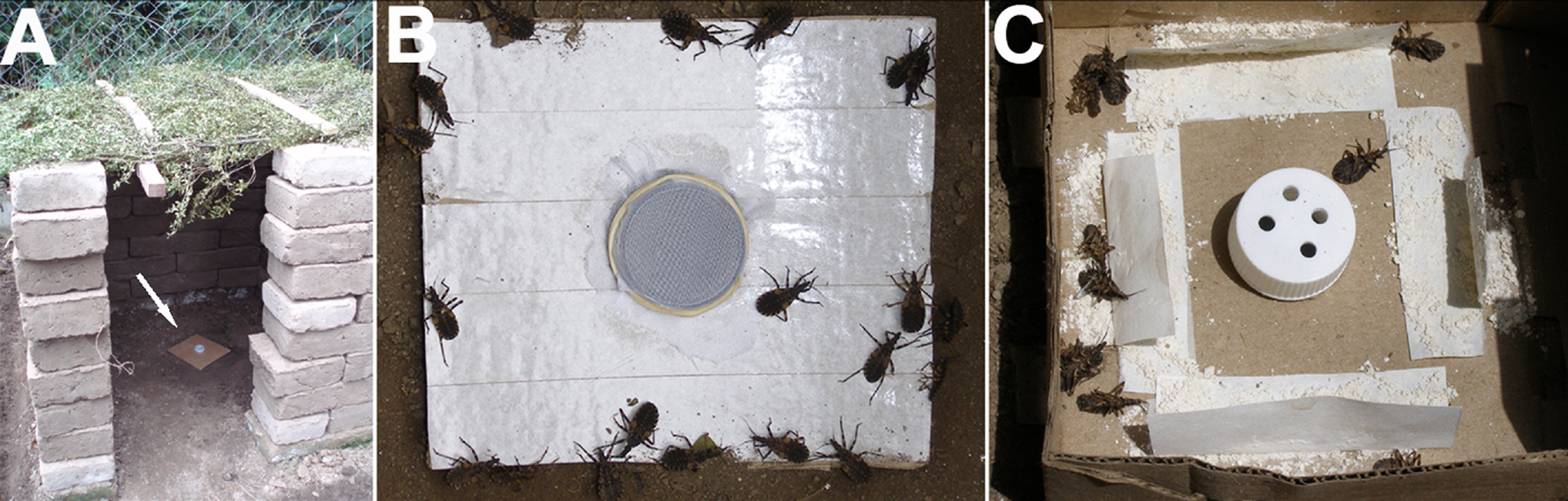

Supplement: Figure S2 — Experimental field assay. A) Experimental house. The arrow shows the attraction trap location. B) Bugs catched on a sticky surface during a 6-h period exposure to the CO2 source. The bottle containing the yeast suspension is buried on the floor; the central opening is covered with muslin and located at the floor level. C) Bugs killed in the “attraction-infection” device; here the CO2-releasing bottle is covered with a perforated lid. Only the bottom side of the trap is shown in the picture. (2.12 MB TIF) [file pntd.0000434.s002.tif]
